# Mevastatin blockade of autolysosome maturation stimulates LBH589-induced cell death in triple-negative breast cancer cells

**DOI:** 10.18632/oncotarget.14868

**Published:** 2017-01-27

**Authors:** Zhaohu Lin, Zhuqing Zhang, Xiaoxiao Jiang, Xinhui Kou, Yong Bao, Huijuan Liu, Fanghui Sun, Shuang Ling, Ning Qin, Lan Jiang, Yonghua Yang

**Affiliations:** ^1^ Department of Pharmacology and Biochemistry, School of Pharmacy Fudan University, Shanghai 201203, China; ^2^ Interdisciplinary Research Institute, Shanghai University of Traditional Chinese Medicine, Shanghai 201203, China; ^3^ Chemical Biology, Roche Pharmaceutical Research and Early Development, Roche Innovation Center Shanghai, Shanghai 201203, China; ^4^ Department of Biological Sciences, Oakland University, Rochester, MI 48309, USA

**Keywords:** breast cancer, mevastatin, HDAC inhibitor, LBH589, autophagy

## Abstract

Histone deacetylase inhibitors (HDACi) are promising anti-cancer agents, and combining a HDACi with other agents is an attractive therapeutic strategy in solid tumors. We report here that mevastatin increases HDACi LBH589-induced cell death in triple-negative breast cancer (TNBC) cells. Combination treatment inhibited autophagic flux by preventing Vps34/Beclin 1 complex formation and downregulating prenylated Rab7, an active form of the small GTPase necessary for autophagosome-lysosome fusion. This means that co-treatment with mevastatin and LBH589 activated LKB1/AMPK signaling and subsequently inhibited mTOR. Co-treatment also led to cell cycle arrest in G2/M phase and induced corresponding expression changes of proteins regulating the cell cycle. Co-treatment also increased apoptosis both *in vitro* and *in vivo*, and reduced tumor volumes in xenografted mice. Our results indicate that disruption of autophagosome-lysosome fusion likely underlies mevastatin-LBH589 synergistic anticancer effects. This study confirms the synergistic efficacy of, and demonstrates a potential therapeutic role for mevastatin plus LBH589 in targeting aggressive TNBC, and presents a novel therapeutic strategy for further clinical study. Further screening for novel autophagy modulators could be an efficient approach to enhance HDACi-induced cell death in solid tumors.

## INTRODUCTION

Breast cancer is the second leading cause of cancer-related deaths among women in the United States, with over 246,660 new diagnoses expected in 2016 and approximately 40,450 deaths [1]. Current clinical therapies include hormone-based agents that directly target estrogen receptor (ER), progesterone receptor (PR), or human epidermal growth factor receptor 2 (HER2) [2, 3]. Triple-negative breast cancer (TNBC) is ER-, PR- and HER2-negative, and accounts for approximately 15% of all breast cancers [3–5]. TNBC is an aggressive subtype that does not respond to ER-, PR-, or HER2-targeted therapies, and TNBC patient prognoses are poor [3, 6].

Histone deacetylase inhibitors (HDACi) induce differentiation, senescence, modulation of immune response, altered angiogenesis, cell-cycle arrest, apoptosis, necrosis, and autophagy in a variety of tumor cells [7–9]. Vorinostat and romidepsin were approved by the FDA for T-cell lymphoma treatment in 2006 and 2009, respectively [10, 11]. Although HDACi are effective as single agents against a subset of hematological tumors, they are less effective in solid tumors [7]. Still, HDACi have shown promising synergistic or additive antitumor effects in combination with other antitumor agents [9, 12–14].

LBH589 is an HDACi shown to suppress TNBC cell growth at nanomolar concentrations [15, 16]. LBH589 treatment reduced tumor load *in vivo* and increased overall survival in treated mice [13, 17]. LBH589 combined with other agents may improve treatment efficacy and provide an attractive therapeutic strategy for TNBC. In this study, we show that the combination of mevastatin [18] and LBH589 inhibits TNBC cell proliferation by downregulating the cell cycle regulator, cyclin D1, upregulating P21 activity, and enhancing apoptosis. We show that mevastatin increases autophagososme formation, but decreases autolysosome maturation, potentiating LBH589-induced TNBC cell death. Our results also demonstrate that cellular stress induced by mevastatin plus LBH589 activates LKB1/AMPK to promote TNBC cell death. This activation inhibited mTOR, p70S6K, and cyclin D1, and induced apoptosis. In addition, treatment reduced Rab7 prenylation, inhibiting autolysosome maturation. Mevastatin plus LBH589 also decreased tumor volume in an *in vivo* TNBC xenograft tumor model. Thus, our results show that mevastatin plus LBH589 is a potentially efficacious therapeutic strategy for treating TNBC.

## RESULTS

### Mevastatin enhances LBH589-induced cell death and autophagy marker expression in human TNBC cells

**Figure 1:**
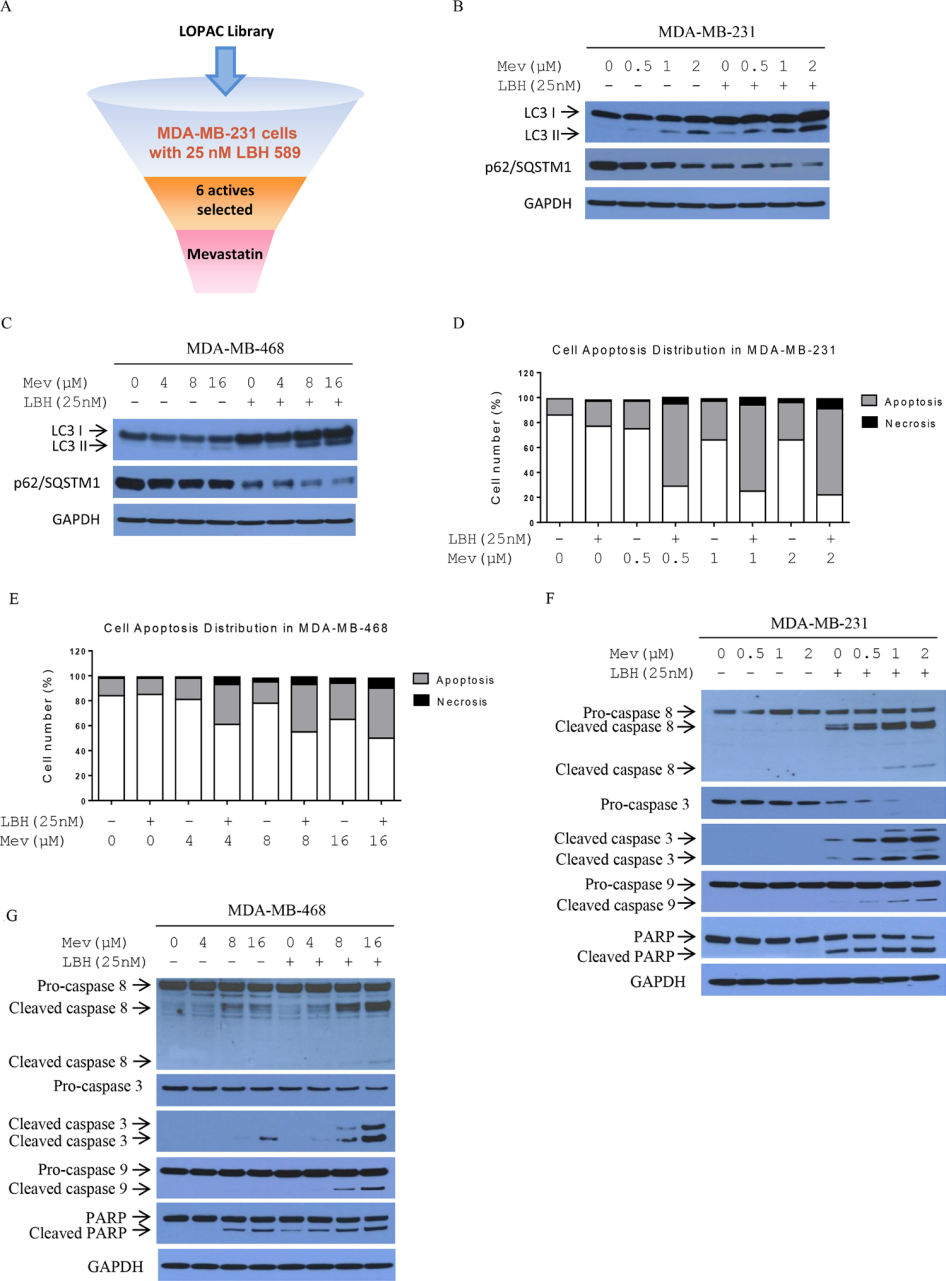
Mevastatin enhances LBH589-induced autophagy and cell death in TNBC cells Screening for suitable partners acting in synergy with LBH589 in TNBC cells (**A**) With or without LBH589 (25 nM), endogenous LC3B and p62/SQSTM1 levels were detected by Western blotting in mevastatin-treated MDA-MB-231 (0, 0.5, 1, 2 μM) (**B**) and MDA-MB-468 cells (0, 4, 8, 16 μM) (**C**) for 24 h. Synergistic cell death induction by mevastatin and LBH589 for 24 h in MDA-MB-231 (**D**) and MDA-MB-468 cells (**E**) followed by FACS analysis. Mevastatin enhanced LBH589-induced apoptosis-related proteins dose-dependently in MDA-MB-231 (**F**) and MDA-MB-468 cells (**G**) as shown by Western blotting.

We used the LOPAC library (Sigma) of 1280 pharmacologically active compounds to identify suitable LBH589-synergistic partners in TNBC cells. Six active compounds were found to increase LBH589 anti-proliferation activity in MDA-MB-231 cells (Figure 1A). The HMGCR (3-Hydroxy-3-Methylglutaryl-CoA Reductase) inhibitor, mevastatin, which catalyzes the critical and rate limiting step in cholesterol and isoprenoid biosynthesis through the endogenous mevalonate pathway [19], effectively sensitized cells to LBH589 at sublethal concentrations (25 nM) (Supplementary Table 1). We then examined the effects of mevastatin and LBH589 on cell growth using three TNBC cell lines: MDA-MB-231, MDA-MB-468 and MDA-MB-453. After 48 h, cell proliferation was measured via CCK8 assay. All cell lines showed dose-dependent responses to mevastatin or LBH589 treatment. All TNBC cell lines treated with LBH589 alone showed similar median inhibitory concentrations (IC_50_) (MDA-MB-231: 36.0 nM, MDA-MB-468: 41.6 nM, MDA-MB-453: 27.1 nM). IC_50_ values for mevastatin in MDA-MB-468 and MDA-MB-453 cells were above 30 μM, and were 8.42 μM in MDA-MB-231 cells. Simultaneous treatment with mevastatin and LBH589 (25 nM) inhibited cell growth more than single agent treatments. With LBH589, mevastatin IC_50_ values improved to 0.75 μM in MDA-MB-231 cells, 8.10 μM in MDA-MB-468 cells, and 17.94 μM in MDA-MB-453 cells (Table 1). In MDA-MB-231 cells, the mevastatin IC_50_ in combination with LBH589 decreased by more than 10-fold compared to mevastatin alone.

**Table 1:** IC_50_ of mevastatin on TNBC cell growth with or without LBH589

	Cell lines	IC_50_ (μM)MEV	IC_50_ (nM)LBH589	IC_50_ (μM) MEV( 25 nM LBH)
Breast Cancer	MDA-MB-231	8.42	36.0	0.75
	MDA-MB-468	> 30	41.6	8.10
	MDA-MB-453	> 30	27.1	17.94

Statins may prevent various human malignancies, including breast carcinoma [19–21]. Through interference with mevalonate and subsequently geranylgeranyl diphosphate (GGPP) biosynthesis, statins inhibit HMGCR cellular GGPP reduction, activating AMPK, further repressing mTOR activity, and promoting autophagy [22–24]. Therefore, we analyzed whether LBH589 plus mevastatin treatment would affect autophagy in MDA-MB-231 and MDA-MB-468 cells. During autophagosome formation, cytosolic LC3-I is cleaved and lipidated to form LC3-II, followed by recruitment to the early phagophore membrane, making LC3-II an excellent autophagosome marker. p62/SQSTM1, normally degraded as part of the autophagy pathway, is a marker for autophagic flux. Similar to our previous LBH589 findings [25], mevastatin alone induced dose dependent LC3-II accumulation and p62/SQSTM1 reduction in both cell lines (Figure 1B–1C). Mevastatin plus LBH589 further increased LC3-II accumulation and decreased p62/SQSTM1 compared to mevastatin alone. Combination treatment induced p62/SQSTM1 degradation. Together, these results indicate greater autophagy induction with combination treatment.

Anti-proliferation activity was further analyzed by annexin-V and PI staining, followed by flow cytometry to measure apoptosis. Mevastatin or LBH589 caused slight increases in apoptotic MDA-MB-231 and MDA-MB-468 cells compared with controls. Combination treatments increased apoptotic and necrotic cells compared to either mevastatin or LBH589 alone in a mevastatin dose-dependent manner. Annexin-V positive MDA-MB-231 and MDA-MB-468 cells increased from 11% and 12% to 69% and 40%, respectively, after 24 h treatment. These results show that combination treatments dramatically induced MDA-MB-231 and MDA-MB-468 cell death (Figure 1D–1E). To assess caspase involvement in combination treatment-induced cell death, cleavage of PARP, and caspase 8, 3, and 9 was examined. Dose-dependent increased cleavage of caspase 8, 3, and 9, and PARP was observed after 24 h of combined treatment, compared to single agents (Figure 1F–1G).

### Mevastatin enhances LBH589-induced G_2_-M phase arrest in human TNBC cells

**Figure 2:**
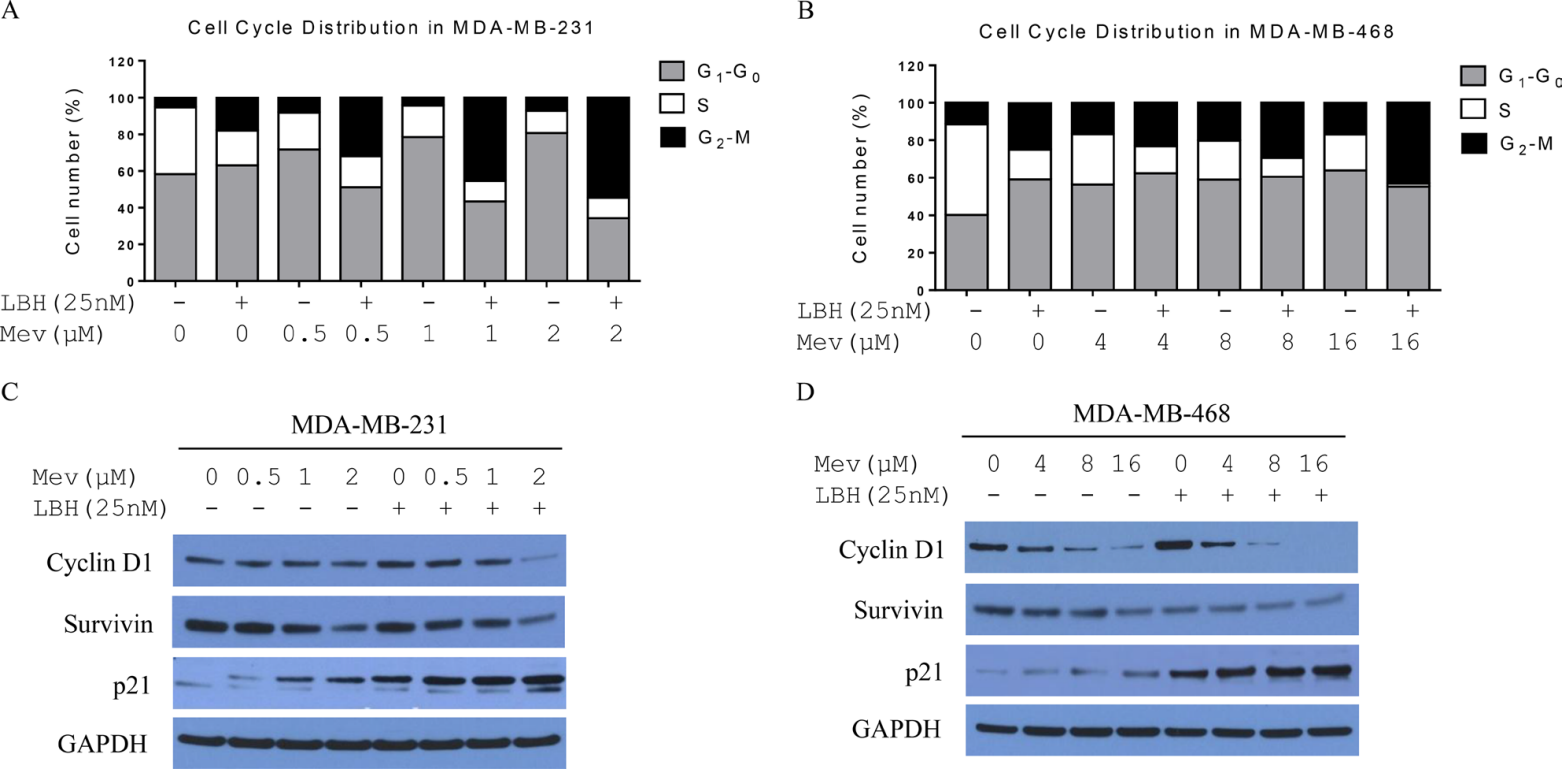
Mevastatin enhances LBH589-induced G2-M arrest in TNBC cells Cell cycle distribution after treatment with or without LBH589 (25 nM) plus mevastatin (0, 0.5, 1, 2 μM in MDA-MB-231 cells (**A**); 0, 4, 8, 16 μM in MDA-MB-468 cells (**B**) for 24 h followed by FACS analysis. Mevastatin regulates cell cycle-related protein expression more efficiently in the presence of LBH589, in a dose-dependent manner. Western blotting of cells treated with or without LBH589 (25 nM) plus mevastatin (0, 0.5, 1, 2 μM in MDA-MB-231 cells (**C**) 0, 4, 8, 16 μM in MDA-MB-468 cells (**D**) for 24 h.

Statins inhibit cancer cell proliferation by arresting the cell cycle at G_1_-S phase and inducing apoptosis via both mevalonate-dependent pathway and -independent pathways [26]. We performed cell cycle analyses via flow cytometry. Mevastatin treatment in MDA-MB-231 cells increased the percentage of cells in G_1_-G_0_ phase from 58% to 81% after 24 h in a dose-dependent manner, and decreased cells in S phase. Treatment with LBH589 (25 nmol/L) for 24 h decreased the percentage of S phase cells from 36% to 16%, and increased cells in G_1_-G_0_ and G_2_-M phases. Combination treatment in MDA-MB-231 cells increased G_2_-M phase arrest from 5% to 55% after 24 h in a mevastatin dose-dependent manner, and decreased cells in S and G_1_-G_0_ phases (Figure 2A). A similar cell cycle distribution was observed in MDA-MB-468 cells (Figure 2B): 24-hour treatment with both mevastatin and LBH589 enhanced G2-M phase arrest (from 12% to 43%) and reduced cells in S and G_1_-G_0_ phases. Cell cycle proteins were analyzed by Western blotting. 24 h combination treatments in MDA-MB-231 and MDA-MB-468 cells downregulated cyclin D1 and survivin, and upregulated p21 in a mevastatin dose-dependent manner (Figure 2C–2D).

### Mevastatin enhancement of LBH589-induced cell death is dependent on caspase 8 activity

**Figure 3:**
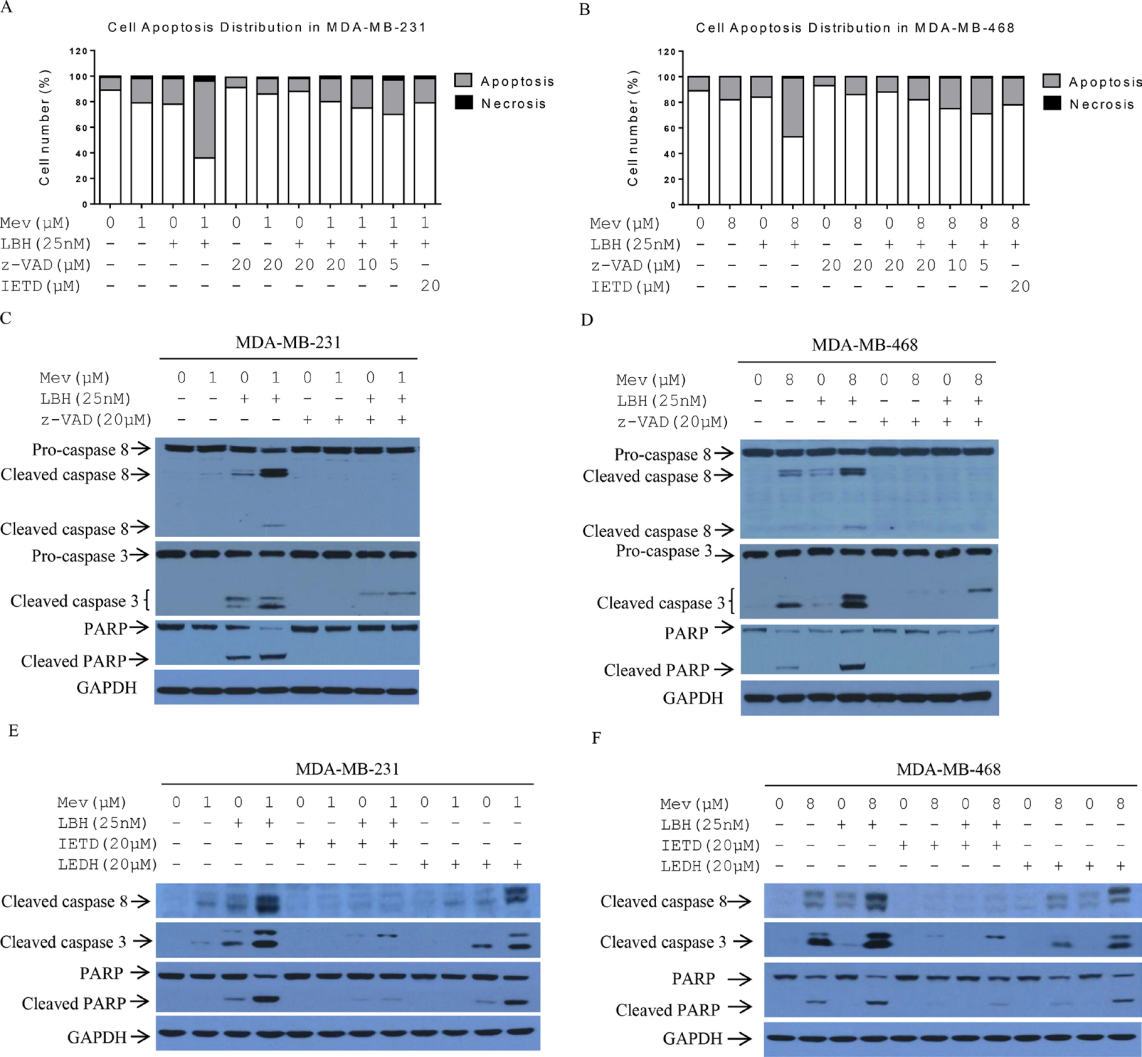
Effect of mevastatin on LBH589-induced cell death MDA-MB-231 and MDA-MB-468 cells were treated with indicated agents for 24 h. Prior to treatment, z-VAD (0, 5, 10, 20 μM) or IETD (20 μM) was added for 2 h to MDA-MB-231 (**A**) and MDA-MB-468 cells (**B**), and apoptosis was assessed via FACS analysis. Cleaved PARP, and caspases 8 and 3 were analyzed via Western blotting with MDA-MB-231 (**C** and **E**) and MDA-MB-468 cells (**D** and **F)**.

To determine whether combined treatment-induced apoptosis was caspase-dependent, we treated cells with the caspase 8 inhibitor z-IETD-fmk (IETD) and the pan-caspase inhibitor z-VAD-fmk (z-VAD), and analyzed apoptotic cells via FACS and immunoblotting. z-VAD treatment alone did not affect Annexin-V binding to cells. However, z-VAD addition to mevastatin (1 μM) and LBH589 (25 nM)-treated cells increased the viable cell fraction from 36% to 80% in MDA-MB-231 cells, and from 53% to 86% in MDA-MB-468 cells (Figure 3A–3B). z-VAD rescued the combination treatment in a dose-dependent manner. Similar to z-VAD, IETD protects both MDA-MB-231 and MDA-MB-468 cells against mevastatin and LBH589-induced apoptosis (Figure 3A–3B). z-VAD and IETD, but not caspase 9-specific inhibitor LEDH, consistently abrogated cleavage of caspases 8 and 3, and PARP induced by 24-h mevastatin (1 μM) and LBH589 (25 nM) treatment in both cell lines (Figure 3C–3F). These results indicate that combination treatment-induced apoptosis is caspase 8-dependent.

### Mevastatin plus LBH589 activates the LKB1-AMPK energy-sensing pathway

**Figure 4:**
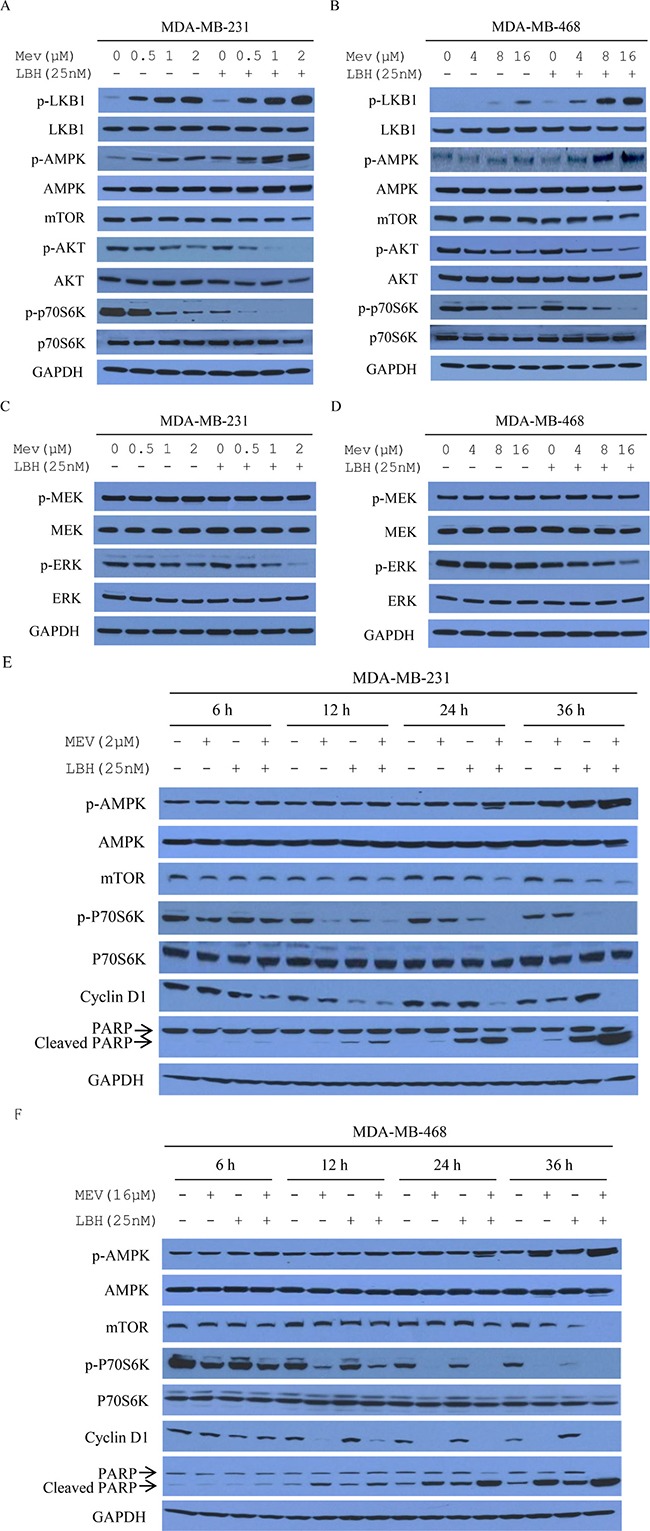
Mevastatin and LBH589 activate AMPK and inhibit mTOR in TNBC cell lines AMPK involvement in the synergistic effects of mevastatin and LBH589 on MDA-MB-231 (**A**) and MDA-MB-468 (**B**) cells for 24 h. Cell lysates were immunoblotted with indicated antibodies. Co-treatment effect on ERK does not depend on Ras in MDA-MB-231 (**C**) and MDA-MB-468 (**D**) cells after 24 h. Cell lysates were analyzed by Western blotting for phosphorylated or total MEK, phosphorylated or total ERK, with GAPDH as an internal control. MDA-MB-231 (**E**) and MDA-MB-468 cells (**F**) were treated with mevastatin plus LBH589 for 6, 12, 24 and 36 h. Lysates were immunoblotted with the indicated antibodies.

We evaluated the effects of combination treatment on intracellular AMPK, AKT, and p42/p44 MAPK (ERK1/2) signaling activation in MDA-MB-231 and MDA-MB-468 cells. 24-h mevastatin and LBH589 treatment increased AMPKα phosphorylation at Thr-172, compared to mevastatin alone, in a dose-dependent manner (Figure 4A–4B). AMPK activation is associated with decreased mTOR and p70S6K activation [27]. Combined treatment decreased mTOR activation, as shown by dose-dependent decreased mTOR expression and reduced phosphorylated p70S6K, compared to single agents and controls (Figure 4A–4B). LKB1 is a tumor suppressor that phosphorylates AMPK at Thr-172 [28]. Its activation was investigated by measuring AMPK phosphorylation at Thr-172 and LKB1 Ser-428 phosphorylation. Combination treatment decreased phosphorylation of AKT and ERK, but not MEK, indicating that the combination effect did not activate Ras/Raf signaling (Figure 4C–4D).

For Western blotting analyses, cells were treated with mevastain (2 μM in MDA-MB-231 cells or 16 μM in MDA-MB-468 cells) plus 25 nM of LBH589 for 6, 12, 24 and 36 h (Figure 4E–4F). Increased AMPK phosphorylation and decreased mTOR were accompanied by reduced downstream p70S6K phosphorylation, decreased cyclin D1, and increased PARP activation in a time-dependent manner.

### Mevalonate pathway inhibition and LKB1/AMPK activation are involved in mevastatin and LBH589-dependent TNBC cell death

**Figure 5:**
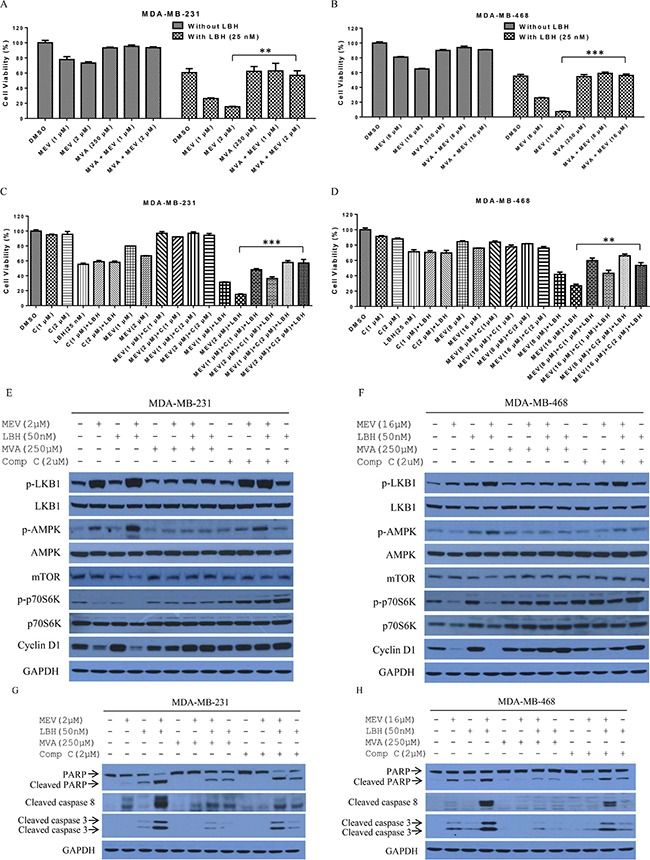
The mevalonate pathway is involved in mevastatin enhancement of LBH-induced TNBC cell death Mevalonate and compound C rescued mevastatin-induced proliferation inhibition in MDA-MB-231 and MDA-MB-468 cells. Prior to mevastatin and/or LBH589 (25 nM) treatment for 48 h, MDA-MB-231 (**A**) and MDA-MB-468 (**B**) cells were incubated with or without mevalonate (250 μM) for 6 h. Alternatively, prior to treatment, MDA-MB-231 (**C**) and MDA-MB-468 (**D**) cells were incubated with or without compound C (1, 2 μM) for 2 h. Mevalonate and compound C blocked LKB1/AMPK signaling in MDA-MB-231 (**E**) and MDA-MB-468 (**F**) cells treated with indicated agents for 24 h. Cell lysates were immunoblotted with indicated antibodies. Mevalonate and Compound C reduced cleavage of apoptosis-related proteins in MDA-MB-231 (**G**) and MDA-MB-468 (**H**) cells. Cleavage of PARP, and caspases 8 and 3 was analyzed by Western blotting. Data are representative of at least three experiments. Student's *t*-test. **P < 0.01; ***P < 0.001.

Mevastatin specifically inhibits the rate-limiting enzyme, HMGCR, in the mevalonate pathway. We found that combination treatment-induced cell proliferation inhibition was reversed by addition of mevalonate. 250 μM of mevalonate (MVA) impaired mevastatin (MEV) anti-proliferation activity in both MDA-MB-231 and MDA-MB-468 cells, and recovered cell viability from 73% and 65% to 94% and 91%, respectively (Figure 5A–5B). Moreover, the mevastatin plus LBH589 synergistic effect was abolished by mevalonate addition (Figure. 5A–5B). Cell viability increased from 14% and 7.8% to 45% and 52%, respectively, in high mevastatin dose (2 μM and 16 μM)-treated MDA-MB-231 and MDA-MB-468 cells. However, mevalonate did not reverse the effect of LBH589 on cell growth in either cell line, indicating that the mevalonate pathway does not participate in LBH589-induced cell death.

In addition to the mevalonate pathway, our results suggested that combination treatment synergy requires AMPK and mTOR signaling. Compound C (C in Figures) is an AMPK inhibitor that blocks AMPK metabolic and anti-apoptotic activities [29]. TNBC cells were treated with compound C, mevastatin or LBH589 alone or in combination for 48 h. Compound C alone or with LBH589 or mevastatin had a marginal effect on cell viability. However, compound C at a dose of 2 μM improved proliferation from 31.4% to 57.9% and 15.0% to 57.1% in MDA-MB-231 cells treated with LBH589 (25 nM) and mevastatin at 1 μM and 2 μM, respectively. At 1 μM, compound C improved MDA-MB-231 cell proliferation from 31.4% and 15.0% to 48.1% and 36.3% in cells treated with LBH589 (25 nM) and mevastatin at 1 μM and 2 μM respectively. 2 μM compound C rescued MDA-MB-468 cell viability after treatment with LBH589 (25 nM) and mevastatin at 8 or 16 μM from 41.8% and 26.9% to 65.9% and 53.5%, respectively, and 1 μM compound C rescued cell viability from 41.8% and 26.9% to 59.9% and 43.2% (Figure 5C–5D), respectively.

Western blotting confirmed that combination treatment induced apoptosis through mevalonate pathway blockage and LKB1/AMPK activation. Combined treatment increased AMPKα phosphorylation at Thr-172 compared to single agents and controls (Figure 4A–4B and 5E–5F). Increased AMPK activation inactivated mTOR, as evidenced by diminished mTOR, p-p70S6K, and cyclin D1 levels in both MDA-MB-231 and MDA-MB-468 cells. Combination treatment-induced AMPK, p70S6K, and LKB1 phosphorylation was abolished by 250 μM of mevalonate. p70S6K phosphorylation was subsequently restored by 2 μM of compound C, which decreased AMPK, but not LKB1, phosphorylation. We also tested whether combination treatment-induced apoptosis could be rescued through the AMPK and mevalonate pathways. Indeed, apoptosis-related protein cleavage was reduced by mevalonate or compound C (Figure 5G–5H). This suggests that both AMPK and mevalonate signaling participate in combination treatment-induced TNBC cell apoptosis.

### Mevastatin abrogates LBH589-induced autophagy in TNBC cells via mevalonate pathway blockade

**Figure 6:**
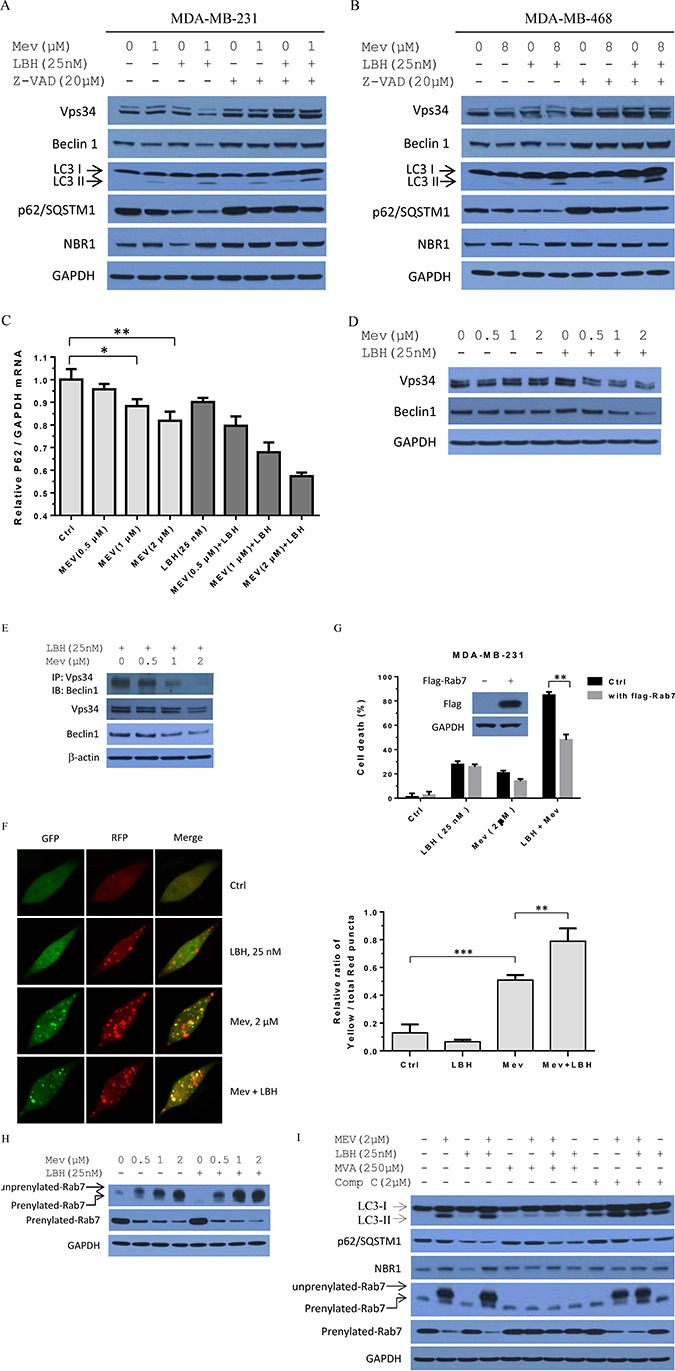
Mevastatin blockade of mevalonate signaling abrogates LBH589-induced autophagy maturation in TNBC cells Prior to mevastatin plus LBH589 (25 nM) treatment for 24 h, MDA-MB-231 (**A**) and MDA-MB-468 (**B**) cells were incubated with or without z-VAD (20 μM) for 2 h. Cell lysates were analyzed by Western blotting for Vps34, Beclin 1, LC3B, p62/SQSTM1, and NBR1, with GAPDH as a loading control. Relative p62/SQSTM1 expression (compared with GAPDH) was analyzed by qPCR. Student's *t*-test. *P < 0.05; **P < 0.01 (**C**). MDA-MB-231 cells were treated with mevastatin (0, 0.5, 1, 2 μM) and/or LBH589 (25 nM) for 24 h. Mevastatin reduced LBH589-dependent expression of Vps34 and Beclin 1 in a dose-dependent manner (**D**). MDA-MB-231 cells were treated with mevastatin (0, 0.5, 1, 2 μM) for 24 h and probed for endogenous Vps34 and Beclin 1. Mevastatin abrogated Vps34-Beclin 1 complex formation dose-dependently in the presence of LBH589 (**E**). MDA-MB-231 cells were treated with mevastatin (0, 0.5, 1, 2 μM) plus 25 nM LBH589. Immunoblot analyses were performed on immuoprecipitates as indicated. tfLC3 stable MDA-MB-231 cells were analyzed by confocal microscopy (**F**). MDA-MB-231_tfLC3 cells were treated with mevastatin (2 μM) and/or LBH589 (25 nM) for 24 h. Representative fluorescence images are shown. MDA-MB-231 cells transfected with flag-Rab7 (or pcDNA3.1) were treated with mevastatin (2 μM) and/or LBH589 (25 nM) for 48 h, and cell proliferation was measured. Student's *t*-test. **P < 0.01; ***P < 0.001 (**G**). Mevastatin with or without LBH589 (25 nM) reduced prenylated Rab7 in a dose-dependent manner (**H**). MDA-MB-231 cells were treated with mevastatin (0, 0.5, 1, 2 μM) and/or LBH589 (25 nM) for 24 hours. Cell lysates were immunoblotted for unprenylated and prenylated Rab7. Prior to treatment with mevastatin (2 μM) and LBH589 (25 nM) for 24 h, MDA-MB-231 cells were incubated with or without mevalonate (250 μM) for 6 h, or with or without compound C (2 μM) for 2 h (**I**). Cell lysates were immunoblotted for LC3B, p62/SQSTM1, and unprenylated and prenylated Rab7, with GAPDH as a loading control.

Consistent with autophagic regulation of apoptosis [30], treatment with LBH589 and mevastatin induced apoptosis in TNBC cells. Mevastatin treatment alone resulted in LC3-II accumulation and p62/SQSTM1 downregulation (Figures 1B–1C and 6A–6B). Combination treatment increased LC3-II and decreased p62/SQSTM1 as compared to either agent alone. This indicates that mevastatin might induce autophagy. However, unlike p62/SQSTM1 downregulation, LBH589-mediated NBR1 downregulation was inhibited by combination treatment, which suggests that mevastatin inhibits rather than induces autophagy. To address this, we assessed Vps34 and Beclin 1, essential molecules in autophagic, vesicle formation. LBH589 and mevastatin co-treatment downregulated Vps34 and Beclin 1 (Figure 6A–6B). This effect was rescued by pan-caspase inhibitor, z-VAD. However, z-VAD abrogates caspase 8 activation and enhances LC3-II accumulation. From these results, we could not conclusively demonstrate that mevastatin (with or without LBH589) induced autophagy.

We performed qPCR to determine whether mevastatin or combination treatment-induced p62/SQSTM1 downregulation was due to transcriptional inhibition. Mevastatin suppressed p62/SQSTM1 expression, and this was more pronounced with co-treatment (Figure 6C). Thus, mevastatin inhibits p62/SQSTM1 transcription, but not through autophagy induction.

Combination treatments showed pronounced dose-dependent Vps34 and Beclin1 reduction observed after 24 h, compared with single agents in MDA-MB-231 cells (Figure 6D). Mevastatin abolished LBH589-induced Beclin1-Vps34 complex formation (Figure 6E). These results, together with mevastatin-induced NBR1 accumulation, suggest that mevastatin may not only block Beclin1-Vps34 complex assembly to reduce the autophagosome formation, but also may impair autophagosome-lysosome fusion for adaptor protein degradation.

To address whether mevastatin disrupted autophagosome-lysosome fusion, we analyzed co-treated MDA-MB-231 cells stably expressing RFP-GFP-LC3 (tfLC3). tfLC3 contains microtubule-associated protein 1 LC3 fused with a pH-sensitive tandem fluorescent fusion protein consisting of monomeric red fluorescent protein (mRFP) and enhanced green fluorescent protein (eGFP). GFP, but not mRFP, signals are quenched in acidic environments after autophagosome-lysosome fusion. In initial autophagic vacuoles, tfLC3 emits green and red fluorescence (merged as yellow), whereas in autophagic vacuoles, only red fluorescence is observed. We investigated the effects of the combined treatment on autophagy maturation by comparing colocalized puncta (yellow dots) and mRFP puncta (red dots). Consistent with previous reports [31, 32], LBH589 increased autophagic flux, shown by decreased GFP puncta and increased RFP puncta (Figure 6F). Notably, mevastatin- or LBH589-treated cells differed: both exhibited green and red puncta, but LBH589 treatment produced fewer yellow puncta, indicating that mevastatin induced autophagosome, but not autolysosome maturation. Co-treatment produced more yellow puncta, indicating that combination treatment might block autophagosome-lysosome fusion to inhibit autophagic flux.

Rab7 is a small GTPase critical in autophagosome/endosome maturation [33, 34], and its activation requires prenylation. To confirm that mevastatin interrupts autophagosome-lysosome fusion mainly via Rab7, cell death was analyzed in Rab7 overexpressing MDA-MB-231 cells treated with LBH589 plus mevastatin. Rab7 overexpressing cells exhibited resistance to combination treatment (Figure 6G). Inefficient fusion with endosomes and/or lysosomes would inhibit autophagosome maturation to amphisomes or autolysosomes, thus inhibiting autophagy. Mevastatin treatment blocked mevalonate signaling, dose-dependently decreasing prenylated Rab7 (Figure 6H). Combination treatment further decreased Rab7 prenylation in comparison to single agents, suggesting that co-treatment enhanced autophagy blockade. Consistent with confocal microscopy observations, our results indicated that combination treatment blocked autophagosome-lysosome fusion.

To address whether mevalonate signaling was involved in mevastatin abrogation of LBH589-induced autophagy maturation in TNBCs, we treated MDA-MB-231 cells with 250 μM mevalonate. Immunoblotting showed that mevalonate, but not compound C (2μM), reversed mevastatin- or combination treatment-induced effects on LC3 II, p62/SQSTM1, NBR1 and prenylated Rab7 (Figure 6I). However, compound C induced LC3 II accumulation [29]. Taken together, our results demonstrated that mevastatin abrogates LBH589-induced autophagy maturation in TNBCs through blockade of mevalonate signaling.

### LBH589 plus mevastatin enhances TNBC death *in vivo* in MDA-MB-231 xenograft mice

**Figure 7:**
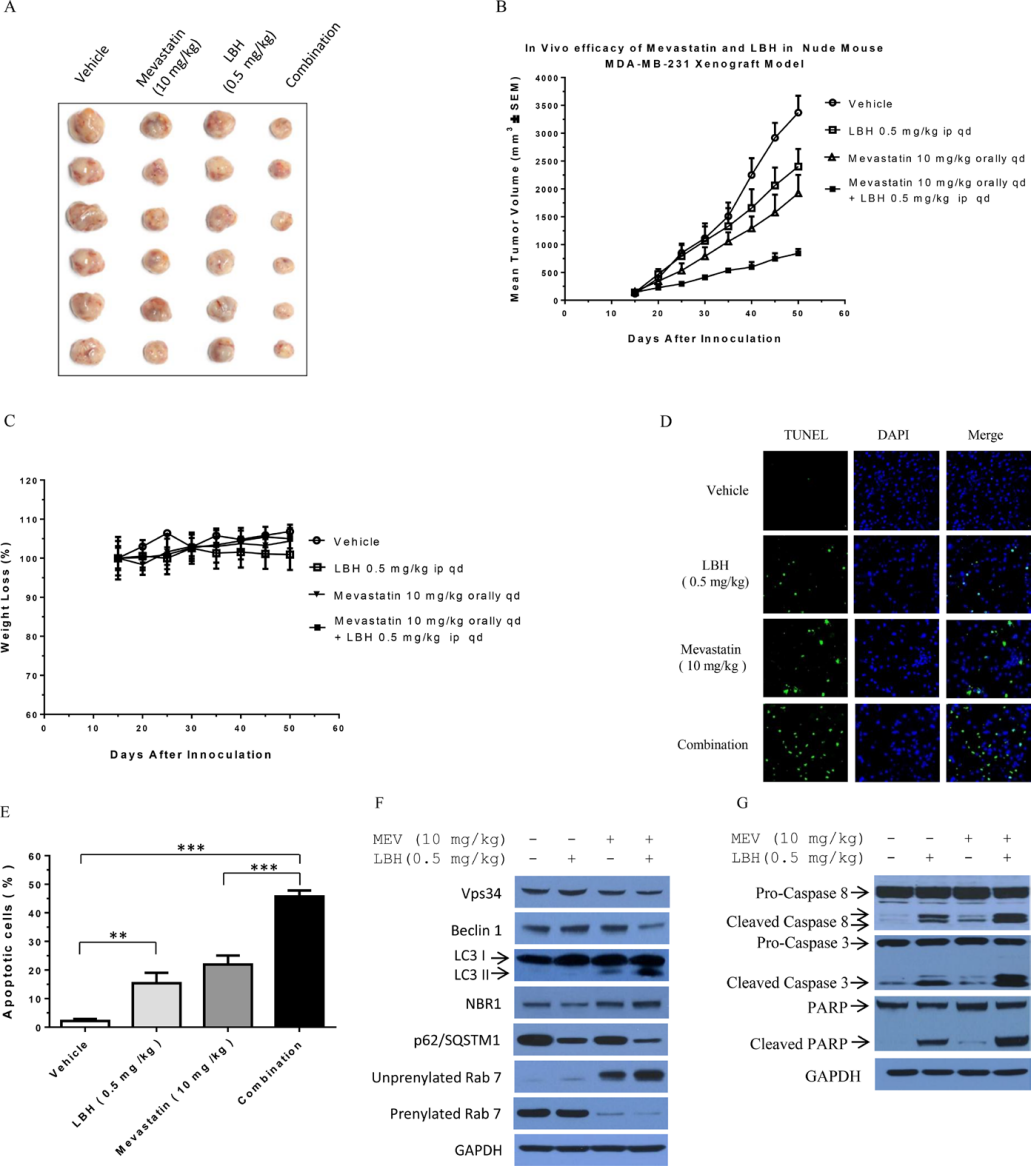
Mevastatin plus LBH589 enhances TNBC cell death *in vivo* in MDA-MB-231 cell xenografted mice Photographs of subcutaneous tumors resulting from indicated treatments in xenografted nude mice (**A**). Tumor-bearing mice were treated with vehicle control, mevastatin (10 mg/kg orally daily), LBH589 (0.5 mg/kg intraperitoneal injection daily), or combination as indicated (**B**). Mouse body weight curves following treatments (**C**). Detection of DNA strand breaks by TUNEL assay in MDA-MB-231 tumors after treatment with vehicle, mevastatin (10 mg/Kg orally daily), LBH589 (0.5 mg/kg intraperitoneal injection daily), or combination (**D**) Green fluorescence indicates TUNEL positive cells. Tumors were stained with DAPI to identify nuclei. Quantitative evaluation of TUNEL positive cells treated with indicated agents (**E**). Five images were analyzed in each group; bars over columns represent means ± SD; Student's *t*-test. **P < 0.01; ***P < 0.001. Immunoblotting analysis of xenograft tumor tissues (**F** and **G)**.

We established an MDA-MB-231 cell xenograft model in nude mice. Mice were treated with vehicle control, mevastatin (10 mg/kg, orally daily), LBH589 (0.5 mg/kg, intraperitoneal injection daily), or mevastatin plus LBH589. Treatments began when tumors reached 100 mm^3^ on average, and tumor growth was measured every five days after treatment initiation. Mevastatin plus LBH589 was more effective in inhibiting tumor growth compared to either mevastatin or LBH589 alone (Figure 7A). Animals treated with mevastatin plus LBH589 exhibited very low tumor growth, with an average final tumor volume of 842 mm^3^, as compared to controls (3369 mm^3^), mevastatin alone (1924 mm^3^), and LBH589 alone (2401 mm^3^), without obvious toxicity (Figure 7B–7C). Mevastatin stimulated LBH589-induced apoptosis in tumor cells, as demonstrated by TUNEL staining. TUNEL staining showed 2.9% (± 1.7) apoptosis in control cells, 21.9% (± 3.2) in mevastatin-treated cells, 15.4% (± 3.6) in LBH589-treated cells, and 45.7% (± 2.1) in co-treated cells (Figure 7D–7E).

Finally, we detected Vps34, Beclin 1, LC3 II, p62/SQSTM1, unprenylated Rab7, prenylated Rab7, caspase 8, caspase 3 and PARP proteins in xenografted tumor samples. Consistent with our cell culture observations, mevastatin plus LBH589 downregulated Vps34, Beclin1, prenylated Rab7 and p62/SQSTM,1 but elevated LC3 II, unprenylated Rab7, and NBR1 (Figure 7F). Combined treatment enhanced cleavage of caspases 8 and 3, and PARP, compared to single agents and controls. This suggests that mevastatin inhibition of autophagy maturation enhances LBH589-induced apoptosis in TNBCs. Thus, combined treatment leads to tumor cell apoptosis through autophagosome-lysosome fusion blockage and subsequent impaired autophagy maturation. LBH589 plus mevastatin may be an effective novel treatment for aggressive TNBC.

## DISCUSSION

HDAC dysregulation in cancer cells leads to gene expression changes and regulates cancer cell viability [12, 35]. Drugs that inhibit HDAC in cancer cells increased survival in mice bearing TNBC cell xenografts [16, 31]. However, HDACi exhibit only limited effectiveness in low concentrations in solid tumors [7, 12]. A potential solution to this limitation is combination therapy. LBH589 has been evaluated in clinical trials in combination with other drugs [8, 11, 15]. Our study employed an unbiased combinatorial screening, including over 1000 drug combinations, to assess synergistic effects of LBH589 with other drugs for TNBC therapeutic application. We found that treatment with LBH589 and mevastatin synergistically reduced MDA-MB-231 and MDA-MB-468 cell proliferation and increased apoptosis (Supplementary Figure 1). Co-treatment decreased tumor growth in *in vivo* MDA-MB-231 xenografts with minimal toxicity.

Statins reportedly exert anti-tumor and anti-proliferation activities *in vitro* and *in vivo*, and the clinical utility of these drugs continues to evolve [36]. Statins may exert anti-cancer effects through decreased mevalonate synthesis, and downregulation of products downstream of mevalonate, including dolichol, geranylpyrophosphate and farnesylpyrophosphate. Recent studies indicate important roles for statins in autophagy: autophagy induction through decreased GGPP, and autophagy blockade through decreased prenylated RhoA [22, 37–41].

We investigated the effects of mevastatin alone or in combination with LBH589 on the initial stage of autophagy, autophagic vesicle formation, and the late stage, autolysosome formation. We employed RFP-GFP-LC3 (tfLC3) fluorescent analysis to investigate the hypothesis that autophagy blockade is involved in the cellular response to co-treatment. A ratio of yellow/RFP-positive puncta = 1 indicated autophagy blockage, while a ratio < 1 indicated abnormally reduced autophagosome-lysosome fusion. The decreased yellow/RFP-positive puncta ratio in LBH589-treated cells confirmed autophagy induction and increased autolysosome formation as previously reported [25]. Our results also demonstrated that mevastatin induced autophagy, but this was partially impaired by reduced autophagosome-lysosome fusion, as indicated by yellow/RFP-positive puncta. In co-treated cells, overwhelming RFP-positive/GFP-positive puncta co-localization indicated disruption of autolysosome maturation and subsequent blockade of autophagy flux.

Rab7 is one of the most important effectors in autophagolysosome maturation, promoting microtubule plus-end-directed transport, and facilitating autophagosome-lysosome fusion [34]. Our results showed that mevastatin or mevastatin-LBH589 co-treatment decreased autophagosome-lysosome fusion through downregulation of prenylated Rab7. This could be reversed by mevalonate addition, or partially rescued by the AMPK inhibitor, compound C, indicating that the mevalonate pathway is involved in autophagosome-lysosome fusion through Rab7 prenylation. Other studies also suggest that lysosomal positioning proteins, such as TFEB (transcription factor EB) and the AAA-type ATPase SKD1, also impair autophagosome-lysosome fusion [42, 43]. Thus, other factors may contribute to the co-treatment autophagosome-lysosome fusion inhibitory effect. Vps34 forms a complex with Beclin 1 in phosphoinositide generation, which is essential for autophagic vesicle formation. Our co-IP results revealed Vps34/Beclin 1 complex formation disruption in mevastatin plus LBH589-treated cells. This blockade inhibits autophagy initiation by reducing autophagosome formation, contributing to disruption of autophagy flux.

**Figure 8:**
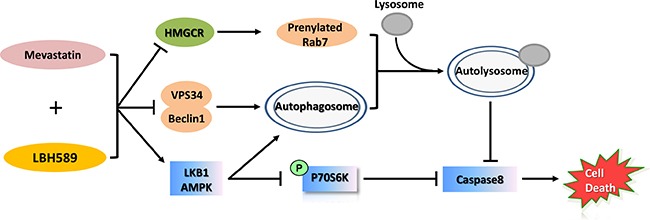
Schematic diagram of the antitumor activities of mevastatin and LBH589 in TNBC This schematic diagram shows that mevastatin increased HDACi LBH589-induced cell death in TNBC cells. Combination treatment inhibited autophagic flux by preventing Vps34/Beclin 1 complex formation and the maturation of autophagosome-lysosome via down regulated prenylated Rab7 as well as the activity of P70S6K by activated LKB1/AMPK signaling, resulting in Caspase8 dependent cell death.

In summary, autophagy blockade was examined as a potential mechanism underlying the synergistic enhancement of anticancer activity via mevastatin-LBH589 combination treatment (Figure 8). We investigated the effects of mevastatin on autophagy and apoptosis. We showed that mevastatin inhibits autophagic flux by abrogating autophagosome-lysosome fusion, and this activity is enhanced by LBH589. Notably, we demonstrated that mevastatin stimulates LBH589-induced apoptosis in TNBC cells. The combination effect was observed both *in vitro* and *in vivo* in a xenograft model. Our results confirm the synergistic efficacy of, and demonstrate a potential therapeutic role for mevastatin plus LBH589 in targeting aggressive TNBC, and offer a new therapeutic strategy for further clinical study. Further screening for novel autophagy modulators could be an efficient approach to enhance HDACi-induced cell death in solid tumors.

## MATERIALS AND METHODS

### Cell lines and reagents

MDA-MB-231, MDA-MB-468 and MDA-MB-453 cells were from the American Type Culture Collection (ATCC). Cells were maintained in RPMI 1640 medium containing 10% fetal bovine serum (FBS; Gibco, Australia) at 37°C in a humidified incubator with 5% CO_2_. Cell Counting Kit-8 (CCK8) was purchased from Dojindo Molecular Tech (CK04-20, Kumamoto, Japan). X-tremeGENE HP DNA Transfection Reagent and *In Situ* Cell Death Detecting Kit were purchased from Roche diagnostic (Indianapolis, USA). LBH589 was purchased from Selleck Chem (Houston, USA). pLenti-C-Myc-DDK-RAB7 (RC201776L1) was purchased from Origene (Rockville, USA). The cDNA encoding mRFP-GFP-LC3 (tfLC3) was excised from ptfLC3 (Addgene) and sub-cloned into the lentiviral vector, pCDH1-MCS1-EF1-puro (System Bioscience). Z-VAD-FMK (FMK001), z-LEDH-FMK and Z-IETD-FMK were purchased from R&D Systems (Shanghai, China). Mevastatin (M2537), Mevalonate (M4667), compound C (P5499), ellipticine (E3380), monoamine oxidase (MAO) inhibitor (Q3251), dequalinium dichloride (D3768), tyrphostin A9 (T182) and DEDA (D8008) were purchased from Sigma (Shanghai, China).

### Cellular proliferation assay (CCK8 assay)

Cells were plated in triplicate and incubated with increasing compound concentrations for 48 h. Proliferation was then measured using a WST-8 (2-(2-methoxy-4-nitrophenyl)-3-(4-nitrophenyl)-5- (2, 4-disulfophenyl)-2H-tetrazolium)-based viability assay (CCK8, Dojindo Molecular Tech). Medium was replaced with fresh WST8- medium (1:10). Cells were incubated 1–2 h, and absorbance at 450 nm was measured using an Envision 2104 microplate reader (PerkinElmer). Compound inhibition rate (IR) was determined using the following formula: IR (%) = (OD_Control_-OD_Mevastatin_)/OD_Control_*100%. For mevastatin and LBH589 combination treatment, IR (%) = (OD_Control_-OD_Mevastatin & LBH589_)/(OD_control_-OD_LBH589_)*100%. The curve of IR against tested compound concentrations was determined with SoftMax Pro software, which was also used to find concentrations corresponding to 50% IR (IC_50_) on the curve.

### Screening of hits in combination with LBH589

MDA-MB-231 cells was seeded in 384 well plates at a density of 12,000 cells per well. Cellular proliferation assay (CCK8 assay) was used for measurement of the cytotoxic effect. An integrated Agilent Biocel 900 System containing direct drive robot (DDR), Liconic CO_2_ incubator, Thermo combi dispenser, liquid handler (Bravo), and a multipurpose reader (Envision, PerkinElmer) enabled high-throughput readout of cell survival after compounds treatment. The substances used in combination with LBH589 were 1,280 pharmacologically active compounds from sigma (LOPAC^®^1280). Cells treated with compounds (10 μM) and/or LBH589 (25 nM) for 48 h followed by cell proliferation detection.

### Cell cycle analysis

MDA-MB-231 and MDA-MB-468 cells were seeded in a 6-well plate at 5.0 × 10^5^ cells/well and treated with mevastatin and LBH589. 24 h later, cells were harvested with trypsin and fixed with EtOH at 4°C for 3 h. Cells were then centrifuged at 1,500 g for 5 min and washed with PBS. Cells were resuspended in RNase/PBS solution (20 μg/ml), incubated at 37°C for 15 min, added to propidium iodide (PI)/PBS solution (20 μg/ml), and incubated at RT for 30 min. Stained cells were analyzed via a FACSCalibur (Becton Dickinson, New Jersey, USA) and data were analyzed using CellQuest Pro software (Becton Dickinson).

### Apoptosis analysis

MDA-MB-231 and MDA-MB-468 cells were seeded in a 6-well plate at 5.0×10^5^ cells/well and treated with mevastatin and LBH589. 24 h later, cells were harvested with trypsin and washed once with pre-cooled PBS. Cells were re-suspended in 500 μl 1× binding buffer, and 5 μL PI and 5 μl annexin-V (Becton Dickinson) were added. After incubation in the dark (room temperature) for 10 min, stained cells were analyzed via a FACSCalibur instrument and CellQuest Pro software.

### Determination of combination effect

MDA-MB-231 and MDA-MB-468 cells were plated in 96-well microplates at 2×10^4^ cells/well. Cells were treated for 48 h with mevastatin from 30 to 0.47 μM with 2-fold dilutions, LBH-589 from 150 to 2.3 nM with 2-fold dilutions, or their corresponding combinations. Synergism after drug treatments was quantitated using the combination index (CI) method in ED50, ED75 and ED90 by Calcusyn software. The Chou and Talalay method was used to calculate combination index (CI). CI > 1, = 1, or < 1 indicates antagonism, additive effect, or synergy, respectively. Each CI ratio is the mean value derived from at least three independent experiments.

### Western blotting analysis

MDA-MB-231 and MDA-MB-468 cells were seeded in a 60 mm dish and treated with mevastatin and LBH589. 24 h later, cells were harvested and lysed by RIPA buffer (150 mM NaCl, 1% NP40, 0.25% deoxycholate and 10 mM Hepes, pH 7.4) containing a protease inhibitor cocktail (Roche Molecular Biochemicals). Protein concentrations were quantified using the bicinchoninic acid (BCA) method (Pierce). 30 μg protein per sample was boiled in loading buffer and electrophoresed in 8–12% gradient SDS polyacrylamide gels (Invitrogen) and transferred onto nitrocellulose membranes. Membranes were probed with antibodies (AKT1 #2967, p-AKT1 #4060, AMPKa #2603, p-AMPKa #2535, Beclin1 #3495, Caspase3 #9662, Cleaved Caspase3 #9661, Caspase8 #9746, Caspase9 #9502, Cleaved Caspase9 #9501, Cyclin D1 #2926, p-Cyclin D1 #3300, GAPDH #3683, MEK1/2 #4694, p-MEK1/2 #9154, mTOR #2972, p-mTOR #2971, p44/42 MAPK (Erk1/2) #9107, p-p44/42 MAPK (Erk1/2) #4370, P70 S6 Kinase #9202, p-P70 S6 Kinase #9205, LKB1 #3047, p-LKB1 #3482, NBR1 #9891, PARP #9542, Rab7 #2094 and Survivin #2808 were purchased from Cell Signaling Technology; p21 and p53 were purchased from R&D; VPS34 #Z-R015 was purchased from Echelon Bioscience; LC3B and β-actin were purchased from Sigma; and p62/SQSTM1 was purchased from MBL), followed by a peroxidase-conjugated immunoglobulin. Western blots were visualized with an enhanced chemiluminescence (ECL) Plus kit (Pierce, ThermoFisher).

### Separation of prenylated and unprenylated proteins

Triton X-114 fractionation was used to separate prenylated and unprenylated Rab7 in whole cell lysates from cultures treated with mevastatin and LBH589. Triton X-114 enables separation of hydrophilic proteins from amphiphilic proteins; therefore, lipophilic, prenylated Rab7 partitions into the detergent-rich phase, whereas unprenylated Rab7 remains in the aqueous phase.

MDA-MB-231 cells were harvested following mevastatin and LBH589 treatment by scraping into medium and pelleting via centrifugation at 1500×g. Cell pellets were washed once in PBS before being lysed in Triton X-114 buffer (20 mM Tris, 150 mM NaCl, pH 7.5, 1% Triton X-114, protease inhibitor cocktail) for 15–20 min at 4°C. Lysates were cleared by centrifugation at 13,000 × g for 15 min at 4°C, and then incubated at 37°C for 10 min. Following centrifugation at 13,000×g for 2 min at room temperature, the top aqueous clear layer was transferred to a new microfuge tube. Buffer without Triton X-114 (20 mM Tris, 150 mM NaCl, pH 7.5, protease inhibitor cocktail) was added to the bottom detergent-rich layer to equalize the volume with the aqueous phase, and protein was electrophoresed on 12% polyacrylamide gels. Gels were analyzed by Western blotting for Rab7 and GAPDH.

### Quantitative real-time PCR (qPCR) analysis

MDA-MB-231 cells were harvested following mevastatin and LBH589 treatment. RNA was isolated with RNeasy Plus Mini kits (QIAGEN) according to the supplier's protocol. Total RNAs (500 ng) was reverse-transcribed in 25 μl using TaqMan^®^ Reverse Transcription Reagents (Applied Biosystems, Inc.).

Each qPCR reaction was performed with 2.5 μL cDNA in 20 μl total volume using the Power SYBR® Green PCR Master Mix. Reaction conditions were as follows: initial denaturation at 95°C for 10 min followed by 40 cycles at 95°C for 10 s, 60°C for 10 s and extension at 72°C for 15 s, then 10 min at 72°C, and 55°C rise to 95°C for melt curves. Primers used included: p62/SQSTM1 (5›-GCA CCC CAA TGT GAT CTG C-3›, 5›-CGC TAC ACA AGT CGT AGT CTG G-3›) and GAPDH (5›-GGA GCG AGA TCC CTC CAA AAT-3›, 5›-GGC TGT TGT CAT ACT TCT CAT GG-3›). All samples were analyzed in triplicate, and reactions were performed using a LightCycler 480 II PCR machine (Roche Diagnostic, Inc.). PCR product amounts were normalized first to GAPDH, and then to levels in control cells as 1.

### Stable MDA-MB-231_tfLC3 cells, and confocal microscopy

Briefly, cDNA encoding mRFP-GFP-LC3 (tfLC3) was excised from ptfLC3 (Addgene, 21074) and sub-cloned into the lentiviral vector, pCDH1-MCS1-EF1-puro (System Bioscience, CD510A-1). Then, the recombinant lentivirus encoding tfLC3 was produced to transduce MDA-MB-231 cells, and 5 μg/ml of puromycin (Invitrogen) was used to select for stable tfLC3-expressing cells, referred to here as MDA-MB-231_tfLC3 cells. Images were acquired with an LSM710 confocal microscope with a 63× objective (Carl Zeiss).

### Immunoprecipitation analyses

After culturing plates for 24 h, 2×10^6^ cells in 100-mm dishes were treated with mevastatin and LBH589. Cell lysates prepared in IP lysis buffer (1% Triton X-100, 150 mM NaCl, 10% glycerol, 30 mM Tris-HCl, pH 7.5) containing protease inhibitors were pre-cleaned by incubation with anti-rabbit IgG-conjugated agarose beads (Sigma, A2909) for 2 h at 4°C, and subjected to immunoprecipitation with anti-VPS34 monoclonal antibodies (Echelon Bioscience, Z-R015). Resulting immune-complexes were washed three times with the lysis buffer and subjected to immunoblotting analyses.

### Transient transfection assay

10×10^5^ MDA-MB-231 cells were seeded in 6-well plates. 24 h later, 2 μg of flag-Rab7 (or pcDNA3.1) was transfected into cells using 6 μl of X-tremeGENE HP DNA transfection reagent (Sigma-Aldrich). 6 h after transfection, cells were re-suspended and seeded in 96 well plates at 2×10^4^ cells/well. After 16 h incubation, cells were treated with mevastatin (2 μM) and/or LBH589 (25 nM) for 48 h followed by cell proliferation detection. Cell death percentage (%) = (OD_Control_-OD_treatment_)/(OD_Control_-OD_blank_)*100%.

### *In vivo* drug activity

MDA-MB-231 (1×10^7^ cells in 0.2 ml PBS) were injected into the left armpits of 5–6-week old BALB/c nu/nu athymic female mice (Sino-British SIPPR/BK Lab. Animal Ltd, China). Tumor volume (mm^3^) was measured with calipers and calculated as (W2×L)/2, where W is width and L is length. When tumor volume reached ~100 mm^3^, mice were randomized into four groups (five mice per group) including control, mevasatin (10 mg/kg orally daily), LBH589 (0.5 mg/kg intraperitoneal injection (ip) daily) and mevastatin (10 mg/kg orally daily) plus LBH589 (0.5 mg/kg ip daily) groups, and treatments were initialized. Sterilized drinking water containing PBS was supplied to control mice. Tumor volumes were recorded every five days. Animals were sacrificed 35 days post-induction. Tumor tissues were collected and subjected to Western blotting analyses for relative protein expression, and TUNEL assay.

### TUNEL assay

TUNEL (terminal deoxynucleotidyltransferase-mediated dUTP nick end labeling) assay was carried out using “*In situ* cell death detection kit, fluorescein” (Roche, USA) according to the manufacturer's instructions. Slides were rinsed twice with PBS. After drying areas around the sample, 50 μl TUNEL reaction mixture was added. After rinsing three times with PBS, samples with fluorescein labels incorporated in nucleotide polymers were detected by fluorescence microscopy. Nuclei were counted by counter staining with 4,6-diamidino-2-phenylindole (DAPI) at excitation wavelength, 350 nm.

### Statistical analysis

All results were expressed as means ± SD of three independent experiments. Statistical analyses were performed using Student's two-tailed *t*-test by Graphpad Prism 6.07 software. *P* < 0.05 was considered significant.

## SUPPLEMENTARY MATERIALS FIGURES AND TABLES


