# Supplementary material for: Mevastatin blockade of autolysosome maturation stimulates LBH589-induced cell death in triple-negative breast cancer cells
Source: Oncotarget. 2017 Jan 27;8(11):17833–48. doi: 10.18632/oncotarget.14868 (PMC5392290; doi:10.18632/oncotarget.14868)
Supplement: Supplementary file 1 [file oncotarget-08-17833-s001.pdf]

Mevastatin blockade of autolysosome maturation stimulates LBH589-induced cell death in triple-negative breast cancer cells

Supplementary Materials

Supplementary Table 1: Hits were identified to notably increase the anti-proliferation activity of 25 nM LBH589 in MDA-MB-231 cells

Cell lines	Hits	IC_{50} (µM)	IC_{50} (µM) (with 25 nM LBH)
MDA-MB-231	Ellipticine	8.65	1.99
	Monoamine oxidase (MAO) inhibitor	8.72	2.59
	Mevastatin	10.13	0.87
	Dequalinium dichloride	3.92	1.52
	Tyrphostin A9	> 60	11.90
	DEDA	> 60	8.14

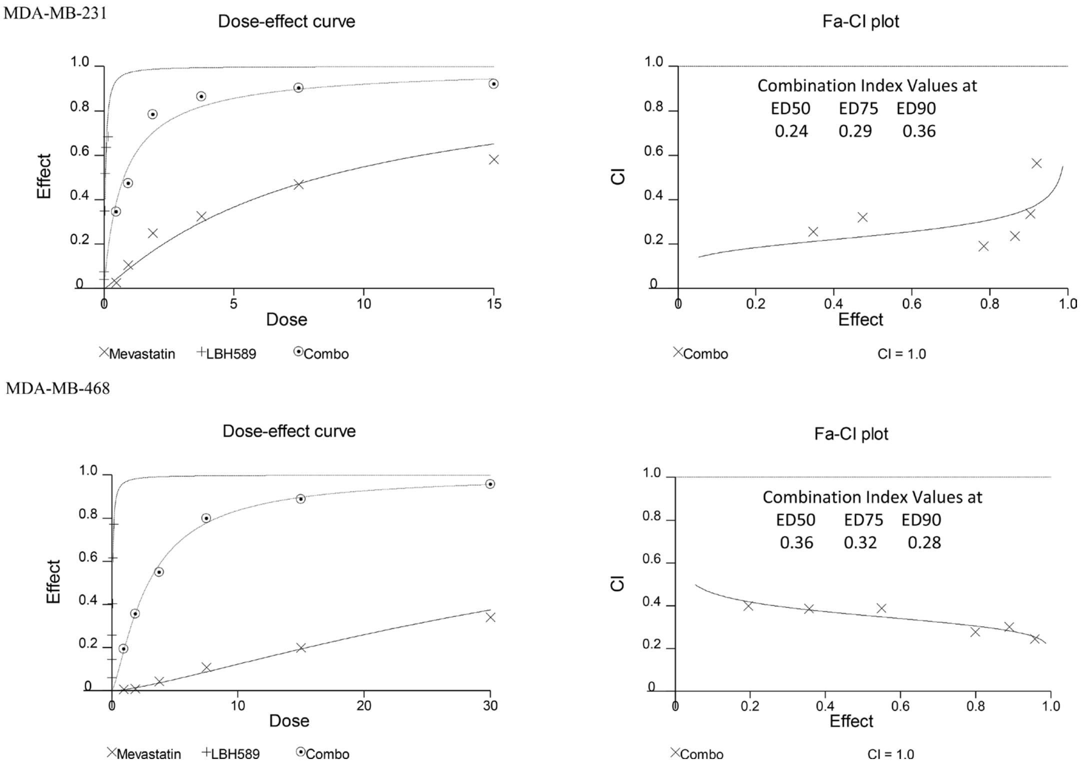

Supplementary Figure 1: Synergistic inhibition of TNBC cell proliferation by Mevastatin and LBH589.
